# Measuring the Effect of the Early assessment Team (MEET) for patients referred to outpatient mental health care: a study protocol for a randomised controlled trial

**DOI:** 10.1186/s13063-024-08028-6

**Published:** 2024-03-11

**Authors:** Camilla Angelsen Kvestad, Ingvild Rønneberg Holte, Solveig Klæbo Reitan, Charlotte S. Chiappa, Gunn Karin Helle, Anne E. Skjervold, Anne Marit A. Rosenlund, Øyvind Watne, Heidi Brattland, Jon Helle, Turid Follestad, Karen Walseth Hara, Katrine Høyer Holgersen

**Affiliations:** 1 Nidelv Community Mental Health Center, Clinic of Mental Health, St. Olav’s Hospital, Trondheim University Hospital, Trondheim, Norway; 2 Department of Mental Health, Norwegian University of Science and Technology, Trondheim, Norway; 3 Clinical Research Unit Central Norway, Faculty of Medicine and Health Sciences, Norwegian University of Science and Technology, Trondheim, Norway; 4 Department of Public Health and Nursing, Faculty of Medicine and Health Sciences, Trondheim, Norway; 5 Norwegian Labour and Welfare Administration Trøndelag, Trondheim, Norway; 6 Department of Psychology, Norwegian University of Science and Technology, Trondheim, Norway

**Keywords:** Community mental health centre, Health service development, Intake assessment, General mental health

## Abstract

**Background:**

Referrals to specialised mental health care (such as community mental health centres; CMHC) have increased over the last two decades. Patients often have multifaceted problems, which cannot only be solved by such care. Resources are limited, and triaging is challenging. A novel method which approaches patients early and individually upon referral to a CMHC—possibly with a brief intervention—is an Early assessment Team (EaT). In an EaT, two therapists meet the patient early in the process and seek to solve the present problem, often involving community services, primary health care, etc.; attention is paid to symptoms and functional strife, rather than diagnoses. This is in contrast to treatment as usual (TAU), where the patient (after being on a waiting list) meets one therapist, who focuses on history and situation to assign a diagnosis and eventually start a longitudinal treatment. The aim of this study is to describe and compare EaT and TAU regarding such outcomes as work and social adjustment, mental health, quality of life, use of health services, and patient satisfaction. The primary outcome is a change in perceived function from baseline to 12-month follow-up, measured by the Work and Social Adjustment Scale.

**Method:**

Patients (18 years and above;* n* = 588) referred to outpatient health care at a CMHC are randomised to EaT or TAU. Measures (patient self-reports and clinician reports, patients’ records, and register data) are collected at baseline, after the first and last meeting, and at 2, 4, 8, 12, and 24 months after inclusion. Some participants will be invited to participate in qualitative interviews.

**Trial design:**

The study is a single-centre, non-blinded, RCT with two conditions involving a longitudinal and mixed design (quantitative and qualitative data).

**Discussion:**

This study will examine an intervention designed to determine early on which patients will benefit from parallel or other measures than assessment and treatment in CMHC and whether these will facilitate their recovery. Findings may potentially contribute to the development of the organisation of mental health services.

**Trial registration:**

ClinicalTrials.gov NCT05087446. Registered on 21 October 2021.

**Supplementary Information:**

The online version contains supplementary material available at 10.1186/s13063-024-08028-6.

## Background

Demand for specialised mental health services is high: the activity at outpatient clinics for specialised mental health care in Norway has tripled since 1998 [1]. Most of this activity is affiliated with the hospitals’ community mental health centres (CMHCs) [2]. Many of the patients referred for outpatient mental health care have complex difficulties, with both mental and somatic ailments in addition to difficulties in social functioning such as education, work, finances, and social support [3, 4]. Individualised measures are often needed to solve the patient’s challenge, and often, interventions other than psychotherapy or advanced psychiatric treatment may be more immediately needed [5]. Traditionally, research and other tasks aiming to improve care in mental health focus on specific diagnoses and an academic approach concerned with how patients fit into diagnostic categories. However, patients do not suffer from diagnoses, but rather symptoms and lack of function; thus, the research literature is seeing a growing interest in knowledge of complex health challenges [6–10] and personalised health care [11]. Complex difficulties are often time-consuming and costly to deal with [12] and today’s effort-driven funding and health service structure based on individual diagnoses seem ill-adapted to the requirements for dealing with these complex issues, which are the reality for most patients [13, 14].

Triage is a well-established term in somatic medicine for assessing urgency and the process of determining clinical needs, but it is used less in mental health services. The assessment of who should be offered specialised treatment is traditionally based on a written referral from the general practitioner (GP); in Norway, this is assessed by the receiving unit in accordance with prioritisation guidelines from the Norwegian Directorate of Health [15]. Depending on the assessed severity, the wait time before the patient first meets their therapist ranges from weeks to months. A wide range of approaches is taken and guidelines vary across different countries [16], illustrating that priority-setting is complex. One study points to possible weaknesses in the prevailing Norwegian model, as it found low agreement between the teams when comparing triage decisions based on written referrals [17]. Another weakness observed in today’s triage process is that there is often no common understanding between the referrer and the hospital specialist regarding the necessity of more specialised treatment [18].

It is a prerequisite that patients referred to a CMHC have significant mental health problems which affect their functional ability in work or everyday life, but the relationship between work and health is complex. It is widely accepted that one of the most dramatic things about mental health problems, often those affecting young people, is that they affect one’s ability to take part in working life [19]. Loss of work is associated with both physical and mental health problems [20–22] in addition to economic consequences, while returning to work appears to be related to improvement in health [23]. A review study indicated the importance of employment for mental health in particular [24]. Consequently, psychiatric symptoms and ailments may develop as a result of not working, studying, or managing daily life in general [25], which may in some cases lead to referral to mental health care. If mental health problems are seen as the result of external stressors, assistance in mastering these external factors may be more appropriate than treatment with psychotherapy or medication. Addressing extra-therapeutic conditions—such as social support and understanding of ongoing life events—in order to increase therapeutic efficacy has been shown by psychotherapy research to be important [26].

The proportion of young people on health-related benefits in Norway is high and has increased over time [27]. Young people without work, education, or training are six times more at risk of reporting feeling depressed and nine times more at risk of reporting ill health. More than half of youth who are ‘not in employment, education or training’ (NEET) have not completed upper secondary school [27]. Various interventions have been attempted at the intersection of disciplines targeting health and work; in Norway, the Return to Work (RTW) programme has shown that it is essential that the treatment focuses not only on symptom reduction but also on improving work capacity and expectations of returning to work [28]. The RTW programme has shown promising results, but it has been criticised for excluding patients with more extensive problems [29]. Another promising intervention is Individual Placement and Support (IPS), which is offered as a supplement in the treatment of both mild and severe mental disorders [30, 31].

While reported loss of function and disability due to mental disorders has increased, the prevalence of serious mental disorders has remained fairly stable in reports from different countries [32–34]. For instance, a recent study from the Netherlands showed an increase in the prevalence of any mental disorder among young adults [35]. In Norway, the latest national reports reveal an increase in self-reported mental symptoms among young adults and students, especially among women [36]; however, while an increase was seen in contact with primary mental health care, this was not the case for specialised mental health care, indicating that the incidence of serious conditions remains constant. This also applies to reported prescriptions, wherein prescribed drugs for the most serious conditions (antipsychotics) were stable, with less than 1% of the population using antipsychotics in the period 2010–2020 [36].

Both a published paper from the European Refinement group and statistics from World Health Organization demonstrate that Norway is in recent years one of the countries with the highest number of public mental health workers per inhabitant [37, 38]; still, waiting lists are increasing, and a shortage of resources is reported. Nevertheless, since both over- and undertreatment of mental illness can be harmful [39, 40], it might be good to evaluate the organisation of services. It is important to ensure that referred patients who are likely to benefit from measures other than medical and/or psychological assessment and treatment are offered such options instead of, before, or in parallel with specialised mental health care. To this end, better integration and coordination of health and other care services have been called for [41]. Interaction with other contributors in health and social services—such as primary health care, community services, or social security services—may be needed to find the right follow-up measures for many of those being referred. This will benefit not only the individual but also the mental health services, by ensuring that there is sufficient capacity for patients with severe mental disorders who need specialised mental health care treatment. From a societal perspective, the health care offered should contribute to reducing social security benefits by increasing the level of functioning.

While organising and handling acute referrals to mental health care have been investigated in a scoping review [42], to our knowledge research on different triage variants in non-acute referrals to CMHCs is still scarce. One Norwegian study investigated the degree to which patient triage based on written referral information corresponded to triage based on consultation with the patient. In many situations, the need determined from a written referral was considered reliable, but in almost half of the referrals (46%), a possible under- or overestimation was seen, indicating a potential risk of incorrect use of resources as well as a risk to patient safety [43]. Another study found that a gateway team operating between primary and secondary mental health care services could reduce inappropriate referrals [44]. One study on health care for children, adolescents, and families suggested that assessment in a triage clinic reduced the frequency of non-attendance at the first appointment, increased the number of cases with shorter treatment courses, and improved interdisciplinary work [45]. One study among students in university health services in the USA concluded that telephone triage enabled rapid clinical intervention at a time when increased efficiency is required [46].

To take on more efficient ways to handle and triage elective referrals to an outpatient clinic, an Early assessment Team (EaT) was established at St. Olav’s University Hospital, Nidelv CMHC, Tiller, in 2017. The purpose was to help patients with an unclear referral receive adapted health care, in line with the national CMHC guidelines and the Health Care Interaction reform [47, 48]. The working method was developed within the frame of the Crisis Resolution Team (CRT), present in the unit since 2008, and drew on experiences from general outpatient clinics over time [49]. The work method in EaT differs from ordinary assessment and follow-up in a general outpatient clinic: it offers a team-based clarification within a few weeks after referral, patients’ problems are understood from a broad perspective (i.e. a biopsychosocial and contextual approach), and the team is organised in a way which supports greater flexibility and accessibility then what is usually possible in a general out-patient clinic. The focus is on immediate collaboration with the community, GP, workplace, etc., and the approach entails identifying patients’ present symptoms and functional strife, rather than establishing an exact diagnosis.

A pilot study evaluating the EaT methodology over its first 2 years indicated a potentially positive effect [5]. More than a thousand patients were referred to EaT who were regarded as in need of service based on written referral. After EaT assessment, two-thirds of these patients were considered to be not in need of further follow-up in a general psychiatric outpatient clinic; this was clarified over the course of 1–3 meetings in 90% of the cases. Less than 20% were re-referred within 6 months, and patients expressed satisfaction with the treatment. However, the effect of the model has not been tested with scientific methods which support generalisation.

## Methods

### Aims and objectives

The aim of this study is to map population characteristics and compare the effect of standard follow-up in general outpatient mental health care (TAU) with that of a new organisation of services, which involves a first meeting with the patient with an Early assessment Team (EaT).

The main hypothesis of the study is that early comprehensive assessment will improve the referred person’s level of function in work and daily life to a greater extent than direct placement on a standard waiting list for traditional diagnostics and individual therapy. Potential effects on mental health, quality of life, use of mental health services, and social security benefits will be examined.

### Trial design

The MEET study is a single-centre, non-blinded, randomised controlled trial with two conditions: treatment as usual (TAU) and EaT. In the TAU condition, the patient has weeks to months of waiting before the initial appointment with a single therapist at the outpatient clinic. The primary objective of this meeting is to investigate the patient’s history for diagnosis and therapy planning. Contact with other services is sequential or parallel and the patient is often the only person talking to the different actors. In the EaT condition, the patient is met shortly after referral (maximum 2–3 weeks) by two therapists, of which at least one is experienced. The overall focus is on the patient’s present problem (symptoms and lack of functioning); in solving this problem, there is a focus on recruiting other collaborators, including social services, workplace, community health care, GP, etc.

The study is designed to investigate the effect of EaT, but it also has a longitudinal and “mixed-methods” study design, including both quantitative and qualitative data. The protocol is designed according to the SPIRIT guidelines for clinical trials [50].

### Research setting

**Fig. 1:**
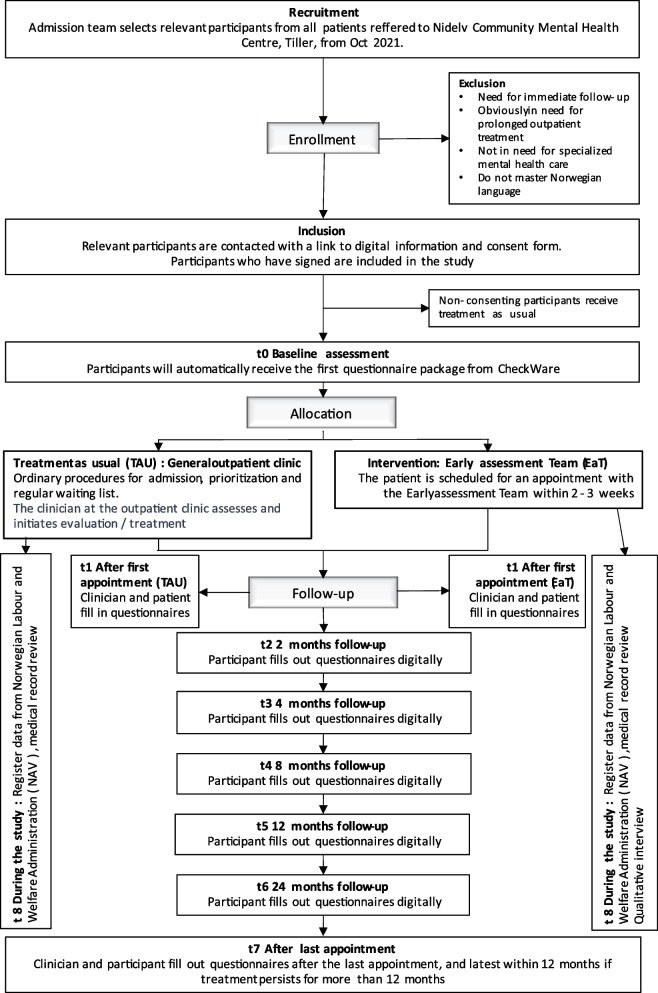
Flow chart providing an overview of the MEET study

The study is conducted at Nidelv CMHC, Tiller, St. Olav’s Hospital, Trondheim University Hospital, Norway. The department is a specialised secondary community mental health hospital for adults. The catchment area is approximately 85,000 inhabitants, and the units’ general outpatient clinic sees 1500–1800 referrals per year. The clinic serves both suburban and rural areas and is a large CMHC for the Norwegian context. Data collection started on 25 October 2021; the duration of data collection will depend on the inclusion rate, continuing until the estimated sample size (*N* = 588) is reached. Figure 1 shows the flow of participants in the study.

### Inclusion and exclusion criteria

All patients referred to the psychiatric outpatient clinic for treatment are considered for inclusion. Patients are adults, specifically 18 years or older. The participants must have enough of a grasp of the Norwegian language that they can understand the written information and give digital or written informed consent. Exclusion criteria include patients who are obviously not in need of specialised health care (e.g. lack of severe symptoms or lack of failure to function), patients who are obviously in need of emergency care, and patients who are obviously in need of long-term psychotherapy or other long-term specialised care. For these groups, it is considered inappropriate to offer an additional level of assessment, as they should obviously either receive further support at the CMHC or have their referral rejected. Patients in need of acute assessment are also excluded, as they cannot be randomised to TAU, which may have a longer waiting time than is considered necessary. Data concerning patients’ mental health diagnoses, previous engagement with mental health services, and physical comorbidities are registered but are not specified as eligibility criteria.

### Interventions

1. Treatment as usual (TAU): Ordinary procedures for admission to an outpatient clinic are followed, in accordance with the Norwegian Directorate of Health’s national prioritisation guide [15]. Referrals are assessed based on severity, and patients are placed on a regular waiting list (usually a waiting period of 1–4 months). They receive their first personal assessment from a therapist in a general outpatient clinic, who decides on further follow-up in accordance with the procedures in the outpatient clinic. There is no standard protocol for what TAU treatment would include, apart from it following national and international standards; each individual therapist is expected to choose the appropriate approach.

In TAU, the patient has been waiting for weeks to months before the first appointment. The patient is met by one therapist—experienced or inexperienced—who may be a psychiatrist, a medical doctor, a psychologist, a psychiatric nurse, or occasionally other health care personnel, such as social workers, physiotherapists, or occupational therapists, most with special training in mental health care. The traditional focus is on building a relationship and exploring the patient’s history to establish a diagnosis and then plan therapy. The first meeting in a standard outpatient clinic is in most cases followed by several assessment and treatment appointments. Therapists vary in how often they collaborate with other professionals. Interaction with other services occurs either sequentially or in parallel, with the patient often being the sole communicator among various stakeholders.

The medical record review performed 12 months after study inclusion registers the different collaboration partners as well as the amount and frequency of health services used.

2) Early assessment team (EaT): The patient is scheduled for their first appointment in the CMHC with the EaT within 2–3 weeks after referral. In the first meeting, the patient meets with two therapists; in most cases, at least one of the therapists is experienced (a psychiatrist or a psychologist specialising in clinical adult psychology), and the other therapist is likely to be a psychiatric nurse or another psychiatrist or a psychologist. A clinical interview is conducted comprising the same topics as in a standard first appointment. Standardised psychometric questionnaires are not usually used in this first meeting: what is most important is the dialogue in which all aspects of life are discussed. Mental symptoms and current ailments are seen from medical, psychological, and contextual perspectives, including the vulnerabilities and strengths reported by patient as well as any sustaining factors from the past and present. Instead of focusing solely on psychiatric diagnosis, the evaluation entails an overall assessment which considers the severity of symptoms and functional impairment experienced by the patient, with a focus on life skills and possibilities.

Different solutions and possibly brief interventions are planned in collaboration with the patient and—if relevant—also with their informal network (i.e. relatives or next of kin) or professional collaborators (i.e. community health care, welfare services, GPs, or others). The team decides whether further assessment or treatment should be provided in the outpatient clinic, or whether any other follow-ups or interventions may be more appropriate; sometimes, a combination of these is applied. The team is flexible in terms of intensity and duration of intervention. Immediate short-term follow-up is possible with the EaT. If the patient’s mental health indicates a need for further standard specialised treatment (TAU after EaT), the patient is transferred to a therapist in the general outpatient clinic. If other means than TAU are expected to sufficiently relieve the patient’s suffering, their contact with the CMHC is ended. When the medical record review is performed 12 months after inclusion, different collaboration partners are registered, along with the amount and frequency of health services used.

The main difference between TAU and EaT is that in the EaT condition, the patient is met early after referral, by two therapists, and the overall focus is on the patient’s present problem. In solving this problem, the focus is on recruiting other collaborators if necessary.

### Procedure: recruitment of participants

After receiving a referral, the admission team decides in the following 10 work days whether the patient meets the inclusion criteria. The procedure is in line with the prioritisation guidelines from the Norwegian Directorate of Health [15]. Eligible patients are invited to participate through an SMS on their mobile phone; the short text message includes a link to more information about the study and the written consent form. If the patient does not reply to the first invitation within three work days, a reminder is sent out automatically. If they have not provided their consent within 5 days after receiving invitation, or if they refuse to participate, the patient is excluded from the study, and a standard letter regarding first assessment is sent. This is in line with the statutory deadlines with which the outpatient clinics must comply.

Immediately after having submitted their written consent, participants receive the first assessment package (T0) via an SMS with a link. Participants are reminded up to two times per dispatch. To reduce drop-out from the study, the second reminder included in this recruitment procedure gives the participants the opportunity to directly ask the researchers questions.

To obtain richer information about the participants’ experiences with EaT, we will invite a subsection of the sample to participate in qualitative interviews, which will focus on their experience with the service they received during and after meeting the EaT. From a given date, 10–15 participants will be recruited from the group which has been randomised to EaT; random selection will be applied to cases in a specified time interval, in which all participants will be informed during their first meeting with the EaT about the possibility of attending a qualitative interview. Participants who return their written consent will be contacted further and will be interviewed by two research assistants and a clinical psychologist who do not work in the EaT. Participants will be interviewed after the first session (if they end their EaT contact after only one meeting) or after the second session if they will have more than one meeting with EaT. In addition, those who continue beyond two sessions will be invited to a follow-up interview when their contact with the EaT has ended.

### Randomisation and blinding

After receiving patients’ consent, the study team randomises them to either the EaT condition or the TAU condition using the web-based randomisation system WebCRF, developed and administered by the Clinical Research Unit at the Faculty of Medicine and Health Science, Norwegian University of Science and Technology, Trondheim, Norway. Due to the design of this clinical study, blinding of the participants and the therapists is not possible.

### Data collection

Data are collected through self-reported questionnaires using an electronic survey system by Checkware® [51]. After giving consent, the participants receive a link to the questionnaires, which are then open for answering for 14 days. Participants may be reminded up to two times per assessment point. The primary assessment points are at enrolment (T0), after the first meeting (T1), and then at 2 (T2), 4 (T3), 8 (T4), 12 (T5), and 24 (T6) months after inclusion, as well as after the last session (T7). If the treatment is not completed at 12 months, the questionnaires from T7 are sent after 12 months. Since waiting times before the first appointment differ in the two conditions, collection points at both 2 and 4 months post-inclusion were added.

Qualified healthcare personnel review the participants’ medical records. In addition, register data related to social security use, income compensation benefits, and sick leave will be collected from the Norwegian Labour and Welfare Administration (NAV). Demographic data are collected as part of the baseline questionnaire package (T0). See Table 1 for an overview of the data collection.

**Table 1:** Schedule of enrolment, interventions, and assessment

	**Study period**										
**Timepoint**	**Enrolment**	**Baseline**	**Allocation**	**Post-allocation**							**Pre-/post-allocation**
		**t**_**0**_		***t***_**1**_ **First meeting**	***t***_**2**_ **2 months**	***t***_**3**_ **4 months**	***t***_**4**_ **8 months**	***t***_**5**_ **12 months**	***t***_**6**_ **24 months**	***t***_**7**_ **Last meeting**	***t***_**8**_ **During the study**
**Enrolment**											
Eligibility screen	X										
Informed consent	X										
Allocation			x								
**Interventions**											
Early assessment team (EaT)			x								
TAU			x								
**Assessments**											
Demographic data		x						X		x	
WSAS		x			x	x	x	X	x		
CORE-OM		x						X	x		
QPR-15		X			x	x	x	X	x		
WHO-5		X			x	x	x	X	x		
AUDIT		X						X	x		
DUDIT		X						X	x		
EQ-5D-5L		X			x	x	x	X	x		
ISI		X			x	x	x	X	x		
WAI				X							
SRS				X							
Questionnaire for therapist				X						x	
CORE-10					x	x	x				
CSQ										x	
Review of hospital medical records		x						x			x
Review of medication use		x						x			x
Register data from Norwegian Labour and Welfare Administration											x
Qualitative interviews											x

*WSAS* Work and Social Adjustment Scale, *WHO-5*, World Health Organization Well-Being Index, *CORE-OM* Clinical Outcome Routine Evaluation, *AUDIT* Alcohol Use Disorders Identification Test, *DUDIT* Drug Use Disorders Identification Test, *EQ-5D-5L* Euro Quality of life, *QPR* Questionnaire Process of Recovery, *SRS* Session Rating Scale, *WAI* Work Alliance Inventory, *CSQ-8* Client Satisfaction Questionnaire

### Assessments

#### Primary outcome measure

The study’s primary outcome is change in perceived function from baseline to 12-month follow-up, measured by the *Work and Social Adjustment Scale* (WSAS; [52]). This is a self-report questionnaire with five items covering the current situation’s influence on work, on home management, on social and private leisure activities, and on relationships with others. The items are scored from 0 to 8, with a total score ranging from 0 to 40; lower scores indicate better adjustment. The scale has been found to have high internal reliability and sensitivity to change. In addition, a positive correlation has been found between WSAS and the grading of depressive symptoms [52]: a mean WSAS score of 25 coincides with major depression (measured by the Hamilton Depression Rating Scale), a mean score of 15.5 corresponds with mild to moderate depression, and a mean WSAS of 6.5 indicates subclinical symptoms. WSAS has also been found to be sensitive in measuring the effect of treatment [53].

#### Secondary outcome measures

##### Self-reported data

Symptoms of psychological distress are measured using the *Clinical Outcome in Routine Evaluation Outcome Measure* (CORE-OM; [54]), a self-administered questionnaire with 34 items concerning the preceding week, mapping four main areas: subjective well-being (four items), problems/symptoms (12 items), social functioning (12 items), and risk to self or to others (six items). All are scored from 0 to 4. The short version, CORE-10 [55], is used instead of the CORE-OM at the assessment points between baseline and the main outcome at 12 months (i.e. T2–T4 + T6). Both CORE-OM and CORE-10 have shown good psychometric properties [55, 56].

Quality of life is assessed using the *World Health Organization Well Being Index* (WHO-5; [57]), one of the most widely used measures of subjective psychological well-being as well as a measure of depression. The scale consists of five simple and non-invasive questions about participants’ well-being, experienced during the preceding 2 weeks, with items rated on a 6-point scale from 0 to 5. A review demonstrated that the questionnaire has excellent psychometric properties [58].

The *Questionnaire about the Process of Recovery* (QPR-15; [59]) is used to capture perceived improvement. The scale was developed in collaboration with service users and has been widely used to capture people’s accounts of their recovery from severe mental illness. The 15-item version has been recommended for use in both routine clinical work and research, and it is scored on a 5-point scale from 0 to 4. Its psychometric properties, specifically its internal consistency, test re-test reliability, and convergent validity, were found to be high [60, 61].

The *Alcohol Use Disorders Identification Test* (AUDIT; [62]) and the *Drug Use Disorders Identification Test* (DUDIT; [63]) examines problematic use of alcohol and drugs during the preceding 12 months. The AUDIT consists of 10 questions assessing the frequency of drinking, average amount of alcohol consumed, worries of others, harm to oneself and others, inability to function without alcohol, and alcohol-induced memory loss; the answers are summed to a total score of 0 to 40 points. The instrument is widely used and gives reliable results [64]. The DUDIT consists of 11 questions, with a total score of 0 to 44 [63]. In addition to items addressing the amount and frequency of use and the signs of dangerous use or addiction, the participant is asked what drugs they use, including illegal drugs and abuse of prescription drugs. The psychometric properties are considered good [65].

The *Euro Quality of life* (EQ-5D-5L; [66]) is used to measure health outcomes and to perform health economic analyses. The scale was originally intended as a supplement to other quality of life measures, but it is increasingly used as an independent instrument. The five component scales consist of questions related to walking, personal care, daily chores, pain or discomfort, and anxiety or depression. In addition, the participant is asked to indicate their subjective state of health on a *Visual Analogue Scale (VAS)* from 0 to 100. The EQ-5D-5L has exhibited excellent psychometric properties across a broad range of populations, conditions, and settings [67].

Patient satisfaction is assessed using the *Client Satisfaction Questionnaire* (CSQ-8; [68]). The form consists of eight questions regarding quality of the service, offers received, and general satisfaction. It is scored using a 4-point scale, *resulting in total scores of 8* to *32*, *with highest scores indicating a greater level of satisfaction*. The CSQ-8 has been widely used in research and is a shorter version of the original 18-item version [69]. The Norwegian CSQ-8 has been reported as having good reliability and construct validity [70].

The *Working Alliance Inventory Short form Revised* (WAI-SR; [71, 72]) is a self-report questionnaire measuring the therapeutic alliance in three domains: agreement with the therapist on the goals of treatment, tasks required to achieve treatment goals, and the quality of the bond established between the patient and the therapist. The 12 items are rated on a 7-point scale ranging from ‘never’ to ‘always’. Higher scores indicate a stronger therapeutic working alliance, and total scores above 36 represent a positive alliance. The WAI-SR has demonstrated adequate psychometric properties [73]. The questionnaire will be administred to the participants at T1, which contains no other secondary measures for patients than the SRS (see below).

*The Session Rating Scale* (SRS; [74]) is used to elicit further information about how the patient experiences their first meeting with the therapist. The four-item self-report measure maps the goals and topics of the conversation, the relationship with the therapist, the therapist’s approach, and a general perception of the meeting. The structure of the SRS is based on a brief visual analogue scale (VAS), where the patient indicates a score on a continuous ten-point scale. The SRS was designed for use by clinicians to assess the therapeutic alliance during treatment, so that the therapist can change their approach or style if the client reports a negative experience. The SRS has solid reliability, sufficient validity, and high feasibility [74]. In this study, the questionnaire is administred to the participants at T1.

Self-reported demographic data at baseline (T0) also includes information about perceived history of cognitive function. Some questions from the *Rapid Assessment of Potential Intellectual Disability* (RAPID; [75]) are used, namely functioning at school, and whether the person has had learning difficulties or received adapted teaching at school. In the demographic data, we also ask if there are problems in different areas of life (e.g. housing, work and education, family relationships, accidents, etc.) and the extent to which the patient and therapist believe this affects the patient’s present functioning and symptoms. The form is based on findings from a Norwegian multicentre study of CRTs [76] and was also used in the pilot study evaluating the first 2 years of the EaT being operational [5].

##### Therapist-reported data

Data from the therapists are reported after the first interview (T1) and at the end of contact or treatment at the CMHC (T7), through questionnaires developed for this study. The aim is to collect information from the therapists about the sessions, the interventions, and their judgement of the patients’ current mental condition, social functioning, and external stressors affecting the current situation. In addition, the therapists fill in the *Health of the Nation Outcome Scales* (HoNOS; [77]), a 12-item scale originally developed to capture the health and social functioning of people with severe mental illness. The scale assesses external factors which may affect function and condition, and each item is scored from 0 to 4.

##### Medical record data

Participants’ medical records are screened to discover contacts with both mental and somatic specialist health services. From these records, ICD-10 diagnosis in previous and current contacts, and prescribed medication 12 months after the first assessment, will be registered.

##### Register data

Register data from the Norwegian Labour and Welfare Administration will be used to obtain an overview of sick leave and work-oriented activity. We are collecting data up to 12 and 24 months after the time of referral, with the options of collecting data up to 5 years after referral and 3 years before. Relevant variables include medical benefits for income compensation—such as sickness benefit, work assessment allowance (AAP), and disability benefit—as well as more direct measures for return to work and work inclusion.

### Qualitative interviews

The qualitative interviews will be individual and semi-structured. The purpose of the first interview (after the first or second session) is to learn more about how the first encounter with EaT was experienced. The interview will focus on topics such as how the interviewee experienced the conversations with and treatment from the EaT, expectations before meeting with the team, and whether the patient felt that they received sufficient help for what they were referred for. In the second interview (for those who receive follow-up with the EaT over three sessions or more), the purpose is to reveal patients’ experiences regarding EaT treatment over some time. The focus will be on how the follow-up was experienced, what was most useful or not useful, and whether the patient received help for what they had been referred for. In a further qualitative sub-project of the study, it may also be relevant to interview external partners (such as GPs, primary mental health care workers, and carers) who have interacted with the EaT in various cases; the implementation of such a sub-project depends on available resources and capacity, and it is not definitively planned.

### Fidelity

Data collection is carried out as part of ordinary clinical activities. Neither the intervention EaT nor the standard follow-up (TAU) is standardised; consequently, measuring fidelity to an established treatment model is not possible. The therapist provides thorough descriptions, number of interventions provided, and potential collaboration with other agencies during treatment; this information is supported by data from the medical records, collected 12 months after inclusion.

### Power and sample size calculation

In previous studies using the WSAS comparing new psychological treatments with TAU, the standard deviations for WSAS scores were observed to be between 7 and 10 [78–80]. For patients with obsessive compulsive disorder or depression, a WSAS total score of 20–40 is reported to indicate moderate to severe psychopathology, a score of 10–20 is associated with failure in functional level but not with severe symptoms, while scores less than 10 are not associated with clinical populations [52]. In addition, a difference of 3.2–4.8 between intervention groups and TAU at the last follow-up was found in the studies mentioned above [78–80].

Both conditions in our study receive interventions from the specialist mental health service; therefore, we chose to define the clinically significant difference between the two groups in mean change on the follow-up measurement at 12 months post-inclusion to be at least three units of the WSAS total score. In order to have a statistical power of 80% to detect a difference in total WSAS scores of three units, with a *t*-test using a standard deviation of ten units for the total WSAS scores, we need 176 in each group (given a significance level of 0.05). If we estimate a drop-out of 40%, we thus need 294 in each group (176/0.6 = 294). This gives a total sample size of 588.

### Statistical analyses

The primary analysis of the outcomes will involve the 12-month time point (change in outcome from baseline to 12-month follow-up). A *t*-test will be performed to compare the randomised groups in terms of the primary outcome (WSAS) as well as the secondary outcomes; this test will be conducted within the framework of linear mixed models (LMM) based on data from all time points. Changes over time will also be estimated from the LMMs. If the assumptions for using LMMs are not met, the analyses can be carried out as separate comparisons at the 12-month follow-up. Due to the large sample, independent-sample *t*-tests can be considered according to the central limit theorem. Non-parametric tests will be used if they are considered more appropriate after the distribution of the data is inspected. The assumption of normal distribution for the residuals of the LMMs will be assessed using visual inspection of histograms and normal QQ plots. Due to the method for parameter estimation (maximum likelihood), the LMMs allow for missing outcome data at one or more occasions as long as data are missing at random (MAR); thus, data from all individuals will be included in the primary analyses.

The sample size is quite large considering that this is a naturalistic clinical trial, and it has been calculated from power considerations for the primary outcome (WSAS). As of 20 September 2023, inclusion of participants had reached 50%; therefore, the research team is analysing baseline data from the self-reports for first half of the participants (*n* = 294), medical record data for the participant before randomisation, and data from therapists at T1 concerning their descriptive assessment of the life areas which are seen as part of the participant’s current situation and data. The goal is to describe the features of the sample as a whole. Depending on the amount of time left before inclusion is complete, the research group may study self-reported data from participants at T1 (WAI-S and SRS) and compare these variables between the groups; if this happens, corresponding analyses will be performed on the entire dataset when available and presented in a later article. No data related to primary or secondary outcomes at T2–T6 will be analysed before inclusion is complete. Baseline characteristics of the randomised groups will also be presented for the full sample after inclusion is complete. *P*-values < 0.05 will be considered statistically significant. No formal adjustments of multiple testing will be performed, due to multiple secondary endpoints, but the issue will be kept in mind when interpreting the results.

### Qualitative methods

In this study, we aim to investigate patients’ experiences with and the various aspects of a service innovation project in mental health care. The qualitative knowledge will complement the findings from the efficacy study and be important in the further development of the programme and any implementation studies. This part of the study will have an exploratory, qualitative design rooted in a phenomenological hermeneutic approach. Phenomenology aims to understand what the world looks like to participants and how they perceive and experience the world [81]. Such a design is particularly suitable for investigating the issues at hand, since experiences with admission interviews and assessments in elective mental health care for adults have so far been little studied. The data will further be analysed using reflexive thematic methodology [82]. In the analyses, we will build on the six steps proposed by Braun and Clarke [82], wherein researchers first familiarise themselves with the data (step 1) by either conducting or listening to interviews or reading transcripts, followed by coding (step 2), theme generation (step 3), and reassessment (step 4), which in turn is followed by defining and naming themes (step 5) and writing up the findings (step 6). In the process, we will strive to use Braun and Clarke’s newer reflexive approach to the method, central to which is taking into account the role of researchers in understanding and analysing data and in developing codes and topics [83].

### Data management

Methods for collecting and storing data have been developed in accordance with the regulations for clinical research at St. Olav’s Hospital. The Data Protection Official at the hospital has been consulted, and a secure area, provided by the hospital’s data department, has been created for data storage. The project and data are owned by St. Olav’s Hospital, Nidelv CMHC. To ensure anonymity, participants are given a non-identifiable study ID at the point of randomisation. The digital service for the self-reports has security level 4.

### Data monitoring and safety

No harmful consequences of participation in this study are anticipated; therefore, no stopping guidelines have been developed, and no interim analyses have been planned. Since participation in the study will not affect the patient’s right to health care and since no invasive procedures are performed, we do not consider that participation entails risk or inconvenience exceeding those possibly existing in standard mental health care. Furthermore, the intervention was investigated thoroughly in a pilot study [5]. As a result, we do not find it necessary to establish a data monitoring committee in this trial, and no auditing procedures has been specified.

Through a new digital medical record system which was implemented in the clinic in 2022, the research group is automatically notified of possible adverse incidents, which are logged as part of the study. Should unexpected or adverse incidents occur or be otherwise reported, the steering group will be informed as soon as the incident is known and will decide on further handling. Participants are all patients at CMHC and receive standard treatment and follow-up according to standard clinical procedures.

### Trial steering committee

The trial steering committee consists of the department head at Nidelv DPS (Svenning), as well as the managers at the three general outpatient clinics at Tiller (Rosenlund, Helle GK, and Skjervold), in addition to the head of the EaT (Chiappa) and the research manager at Nidelv DPS (Helle J). The decision-making body consists of the steering committee and the principal investigator (Holgersen), the associate investigator (Reitan), and the Ph.D. candidates (Kvestad and Holte). The researchers collaborate on a daily to weekly basis with all other working members of the study (clinicians, research assistants) in order to maintain correct recruitment and information gathering, as well as implementation and compliance.

## Discussion

This protocol describes a randomised controlled study which aims to determine the effect of a novel way of assessing newly referred patients to outpatient specialised mental health care.

A better organisation of the system to assess who does and who does not need further assistance from mental health care may likely lead to greater capacity for those who need the specialist service. If the model gives rise to a secure, sustainable organisation of services, it should be broadly implemented; similarly, if the model reveals important weaknesses, these should be shown and addressed, or else the model may need to be adapted or ignored.

### Strengths and limitations

The study is conducted in a highly naturalistic setting, with few exclusion criteria. Participation is not diagnosis-specific, and the study can be described as having a transdiagnostic focus on what may be useful when people are referred to specialised mental health care with complex mental health problems. The entire study and the tested working principles rise from the needs and ideas of clinicians (bottom-up). Therefore, the results will be directly applicable, and the intervention can easily be implemented in future clinical operations if the results show that this is appropriate. This makes the study highly relevant to the field. In addition, the relatively high number of participants and the RCT design add to the study’s strengths.

One obvious limitation of this study is that no fidelity testing can be applied, as the clinicians will continually assess the patient’s/participant’s needs. The study is conducted at only one treatment site; accordingly, its generalisability to other units may be limited. On the other hand, this limits unnecessary variation in data material, which adds to the study’s strengths. Another limitation might be the workload involved in filling out the assessments, which may be demanding for the participating patients and thus may result in unsubmitted questionnaires, missing data, and some potentially choosing to withdraw from the study. However, the opportunity to gain knowledge is considered to exceed the expected load on participants.

## Trial status

This publication is based on protocol version 1 (ClinicalTrials.gov publication date, 21 October 2021). Recruitment for the trial began on 25 October 2021 and is expected to be completed by 2024.

### Supplementary Information

**Supplementary Material 1.**

## Data Availability

The trial dataset will not be made publicly available, as this is clinical information owned by the hospital. The trial was registered in Clinical Trials before recruitment, and the present protocol adds to the open nature of the study.

## Abbreviations

RCT: Randomised controlled trial
REK: Regional Ethics Committee
CMHC: Community mental health centre
EaT: Early assessment Team
TAU: Treatment as usual
MEET: Measuring the Effect of the Early assessment Team
RHA: Central Norway Regional Health Authority
NTNU: Norwegian University of Science and Technology
NEET: Not in Employment, Education or Training
RTW: Return to Work
IPS: Individual Placement and Support
CRT: Crisis Resolution Team
GP: General Practitioner
WSAS: Work and Social Adjustment Scale
WHO-5: World Health Organization Well-Being Index
CORE-OM: Clinical Outcome Routine Evaluation Outcome Measure
CORE-10: Clinical Outcome Routine Evaluation Outcome Measure, short version
AUDIT: Alcohol Use Disorders Identification Test
DUDIT: Drug Use Disorders Identification Test
EQ-5D-5L: Euro Quality of life
QPR: Questionnaire Process of Recovery
SRS: Session Rating Scale
VAS: Visual Analogue Scale
WAI: Work Alliance Inventory
CSQ-8: Client Satisfaction Questionnaire
RAPID: Rapid Assessment of Potential Intellectual Disability
HoNOS: Health of the Nation Outcome Scales
NAV: The Norwegian Labour and Welfare Administration
AAP: Work assessment allowance
LLM: Linear Mixed Model
